# From schizotypy to psychosis: is it a natural continuum?

**DOI:** 10.1192/j.eurpsy.2024.1366

**Published:** 2024-08-27

**Authors:** M. A. Andreo Vidal, M. B. Arribas Simón, M. Calvo Valcárcel, M. P. Pando Fernández, P. Martínez Gimeno, M. D. L. Á. Guillén Soto, B. Rodríguez Rodríguez, N. Navarro Barriga, M. Fernández Lozano, M. J. Mateos Sexmero, C. De Andrés Lobo, M. D. C. Vallecillo Adame, T. Jiménez Aparicio, Ó. Martín Santiago, A. Monllor Lazarraga, M. Ríos Vaquero, L. Rojas Vázquez, L. Sobrino Conde, A. Apario Parra, G. Lorenzo Chapatte

**Affiliations:** Psychiatry, Hospital Clínico Universitario de Valladolid, Valladolid, Spain

## Abstract

**Introduction:**

Schizotypal personality is a condition suffered by 4% of the population. It is defined by presenting interpersonal, behavioral and perceptual features similar to the clinical features of psychotic disorders, such as schizophrenia, in less intensity and dysfunctionality, but at risk of reaching psychosis.

**Objectives:**

Presentation of a clinical case about a patient with premorbid schizotypal personality traits presenting with an acute psychotic episode.

**Methods:**

Literature review on association between schizotypal personality and psychosis.

**Results:**

A 57-year-old woman with a history of adaptive disorder due to work problems 13 years ago, currently without psychopharmacological treatment, goes to the emergency room brought by the emergency services due to behavioral alteration. She reports that “her husband and son wanted to sexually abuse her”, so she had to run away from home and has been running through the streets of the town without clothes and barefoot.

Her husband relates attitude alterations and extravagant behaviors of years of evolution, such as going on diets of eating only bread for 40 days or talking about exoteric and religious subjects, as believing that the devil got inside her husband through a dental implant. He reports that these behaviors have been accentuated during the last month. She has also created a tarot website, and has even had discussions with several users. She is increasingly suspicious of him, has stopped talking to him and stays in his room all day long, with unmotivated laughter and soliloquies.

It was decided to admit him to Psychiatry and risperidone 4 mg was started. At the beginning, she was suspicious and reticent in the interview. As the days went by, communication improved, she showed a relaxed gesture and distanced herself from the delirious ideation, criticizing the episode.

**Conclusions:**

In recent years, there has been increasing interest in understanding the association between schizotypy and serious mental disorder. Several theories understand schizotypy as a natural continuum of personality that reveals genetic vulnerability and that can lead to psychotic disorder when added to precipitating factors. Other theories define schizotypy as a “latent schizophrenia” where symptoms are contained and expressed in less intensity.

Around 20% evolves to paranoid schizophrenia or other serious mental disorders. It is complex to distinguish between those individuals in whom schizotypy is a prodrome and those in whom it is a stable personality trait. To date, studies applying early psychotherapeutic or pharmacological interventions have had insufficient and contradictory results, and the follow-up and treatment of these individuals could be a stress factor and a stigma. Some studies are looking for reliable markers of evolution to schizophrenia in order to establish adequate protocols for detention, follow-up and treatment.

**Disclosure of Interest:**

None Declared

